# The Principal Salivary Gland Is the Primary Source of Digestive Enzymes in the Saliva of the Brown Marmorated Stink Bug, *Halyomorpha halys*

**DOI:** 10.3389/fphys.2019.01255

**Published:** 2019-10-11

**Authors:** Sijun Liu, Bryony C. Bonning

**Affiliations:** ^1^Department of Entomology, Iowa State University, Ames, IA, United States; ^2^Department of Entomology and Nematology, University of Florida, Gainesville, FL, United States

**Keywords:** *Halyomorpha halys*, protease, nuclease, stink bug, salivary gland, gut, sheath saliva, watery saliva

## Abstract

The brown marmorated stink bug, *Halyomorpha halys*, is an invasive, phytophagous stink bug of global importance for agriculture. Tissue-specific transcriptomic analysis of the accessory salivary gland, principal salivary gland (PSG) and gut resulted in identification of 234 putative protease and 166 putative nuclease sequences. By mapping the previously reported proteomes of *H. halys* watery saliva (WS) and sheath saliva to protein sequences translated from the assembled transcripts, 22 proteases and two nucleases in the saliva were identified. Of these, 19 proteases and both nucleases were present in the WS. The majority of proteases and nucleases found in WS were derived from the PSG, in line with ultrastructural observations, which suggest active protein synthesis and secretion by this tissue. The highly transcribed digestive proteases and nucleases of *H. halys* were similar to those of the southern green stink bug, *Nezara viridula*, indicating that these pentatomid stink bugs utilize a similar suite of proteases and nucleases for digestion of plant material. The comprehensive data set for the *H. halys* salivary glands and gut generated by this study provides an additional resource for further understanding of the biology of this pestiferous species.

## Introduction

The family Pentatomidae is comprised of 896 genera and 4,722 species of stink bugs (Rider, 2011), and includes multiple species that are significant pests of agriculture on a global scale (Panizzi et al., 2000). The pestiferous species include the brown marmorated stink bug, *Halyomorpha halys*, the southern green stink bug (SGSB), *Nezara viridula*, the green stink bug, *Acrosternum hilare*, and the brown stink bug, *Euschistus servus*. Management challenges are posed by their high reproductive capacity and by the development of resistance to the classical chemical insecticides used for suppression of stink bug populations (Leskey et al., 2012). Further complications are caused by the wide host range of many stink bug species with damage resulting from the feeding of both nymphs and adults (Bergmann et al., 2013; Panizzi, 2015).

*H. halys*, is an East Asian species that spread into Europe and North America. First detected in the United States in the 1990s (Lee et al., 2013), *H. halys* has spread to most states, and is a serious pest in agriculture in addition to being a nuisance when overwintering inside homes and businesses (Biddinger et al., 2014; Leskey and Nielsen, 2018). *H. halys* can feed on more than 120 host plants (Bergmann et al., 2013; Haye et al., 2015), with the ability to feed on multiple plants important for development and survival. *H. halys* has caused dramatic losses in apple, peach, corn, peppers, tomatoes, and soybean (Biddinger et al., 2014). Management is primarily via chemical control (Kuhar and Kamminga, 2017) and pheromone-based attractants show promise (Weber et al., 2014; Rice et al., 2018).

Stink bugs feed by inserting their piercing-sucking mouthparts (stylets) into plant tissues (phloem or xylem) either by salivary sheath feeding or by physically rupturing cells (Backus et al., 2005; Lucini and Panizzi, 2018a, b). For salivary sheath feeding on phloem or xylem vessels, stink bugs secrete gelling or SS to form a flange at the site of penetration into the plant and a stabilizing sheath around the stylets (Lucini and Panizzi, 2018b). For both feeding strategies, WS is released to digest cell contents, and the predigested plant material is subsequently ingested. Further digestion occurs within the gut. The complementary digestive enzymes in the saliva and gut tissues result in efficient metabolic use of ingested plant material by the stink bug (Lomate and Bonning, 2016, 2018; Liu et al., 2018).

The WS produced by hemipteran insects was hypothesized to contain enzymes required for the digestion of plant proteins (Miles, 1964, 1972; Moreno et al., 2011). The *H. halys* WS and SS proteomes revealed distinct protein compositions (Peiffer and Felton, 2014), but few proteases and nucleases were identified from this study as genomic resources for *H. halys* were limited at the time. Since then, genomic resources for *H. halys* have significantly improved (The i5k Initiative, 2017). Two transcriptome studies of *H. halys* that characterized transcriptomes of whole insects at various developmental stages using different bioinformatics tools have been reported (Ioannidis et al., 2014; Sparks et al., 2014). In addition, we characterized the digestive proteases and nucleases of the southern green stink bug, *N. viridula*, at the biochemical, transcriptomic, and proteomic levels with a focus on the salivary gland (ASG and PSG) and gut tissues (Lomate and Bonning, 2016; Liu et al., 2018). The annotated *H. halys* genes provided a blueprint for our *N. viridula* transcriptomic and proteomic analyses. We also conducted a biochemical analysis of digestive enzymes in the same tissues of *H. halys* (Lomate and Bonning, 2018). These studies reinforced the complementary roles of the gut and salivary glands in producing different sets of enzymes for efficient digestion of plant materials by stink bugs.

The goals of this study were to (1) assess whether common digestive enzymes are used by different phytophagous stink bugs, and (2) determine the relative roles of the ASG and PSG in production of salivary enzymes. To this end, we conducted transcriptomic analysis of the *H. halys* salivary gland (ASG and PSG) and gut tissues. Transcripts for putative digestive proteases and nucleases were identified and relative transcription levels determined. Transcripts for digestive enzymes were then translated, and *H. halys* and *N. viridula* proteomes mapped to the translated sequence dataset. This analysis allowed for further identification of secreted proteins including proteases and nucleases in the WS and SS. In addition to providing for comprehensive characterization of *H. halys* digestive enzymes, this study also allowed for comparison of enzyme types and transcription levels by tissue with those of *N. viridula*.

## Materials and Methods

### Tissue Collection and RNA Isolation

The ASG, PSG, and gut tissues were dissected from one hundred *H. halys* adults. Tissues of each type were pooled and directly homogenized in Trizol reagent (Invitrogen, Carlsbad, CA, United States). Total RNA was isolated from the tissues according to the manufacturer’s directions. The quality and integrity of the RNA samples was determined using a 2100 Bioanalyzer (Agilent Technologies, Santa Clara, CA, United States) and agarose gel electrophoresis.

### Preparation of cDNA Libraries and Illumina Sequencing

Three mRNA-Seq libraries derived from ASG, PSG, and gut were prepared by using the TruSeq RNA kit (Illumina Inc., San Diego, CA, United States) according to the manufacturer’s instructions. Single-end sequencing was performed using the Illumina HiSeq2500^TM^ (Illumina Inc., San Diego, CA, United States) to generate 100 base reads. Construction of the mRNA-Seq libraries and sequencing were performed by the DNA Facility at Iowa State University using standard procedures.

### Sequence Assembly, Data Analysis, and Bioinformatics

The quality of the raw sequence reads was examined using FASTQC^1^ (Wingett and Andrews, 2018). Low quality reads and bases were trimmed using the FASTQ Quality Filter of the FASTx-toolkit^2^. Transcripts were *de novo* assembled using Trinity assembler (v2.1.1) (Haas et al., 2013). Reads per kilobase million (RPKM) were estimated using the “align_and estimate_abundace.pl” of Trinity software with RSEM (RNA-Seq by Expectation-Maximization) methods (Li and Dewey, 2011). Contigs of ≥200 nt were selected for further analysis. Sequence annotation for the assembled transcripts was performed using the BLASTx search engine against the NCBI non-redundant (nr) protein database. Gene ontology (GO) annotation of transcripts was achieved by use of the BLAST2GO software^3^ (Conesa et al., 2005). Protease and nuclease transcripts were identified based on the identity of top hits from BLASTx analysis. Transcripts (≥300 nt) with top hits of protease, proteinase, peptidase, or nuclease from the BLAST search were selected for further analysis.

The transcripts of putative protease and nuclease enzymes were further verified by BLASTp annotation. RNA and protein sequence alignments and other analyses such as sequence similarity and identity, were preformed either by use of the multiple sequence alignment tool (Clustal Omega^4^) (Sievers and Higgins, 2018) or by use of BioEdit^5^.

Identification of conserved domains and putative function associated with the enzymes was conducted using the BLAST domain search. Putative enzymes with functions in the mitochondrion or with tRNA activity were excluded from analysis. The sequences of the selected transcripts were checked individually, and unique transcripts, including those with incomplete sequences, were determined by sequence analysis. The presence of a potential signal peptide encoded by full-length protease sequences was predicted using the web-based SignalIP 4.1 server^6^ (Nielsen, 2017; Almagro Armenteros et al., 2019).

Raw sequence data were submitted to NCBI Sequence Read Archive (SRA BioProject: PRJNA560285).

### Mapping of Putative Protein Sequences to Proteomic Profiles Derived From *H. halys* and *N. viridula*

To identify putative proteases and nucleases expressed in the ASG, PSG and gut of *H. halys*, the putative protein sequences of ≥100 amino acids (aa) were translated using TransDecoder software^7^. Acquisition of proteomic data for *H. halys* WS and SS has been described previously (Peiffer and Felton, 2014) and these data were kindly provided for the current study by Drs. Michelle Peiffer and Gary Felton, Department of Entomology, Pennsylvania State University, United States. Methods for mapping the *H. halys* proteomics data and previously published *N. viridula* gut and salivary gland proteomics data (Lomate and Bonning, 2016) to putative protein sequences translated from assembled *H. halys* transcripts were adapted from Liu et al. (2018).

### Construction of Phylogenomic Trees

Proteases and nucleases identified from the salivary proteomes of *H. halys* were aligned to the NCBI nr database by BLASTp. For gene hits derived from insects and other arthropod species, full-length protein sequences were selected for investigation of their phylogenomic relationships. Protein sequences were aligned by MAFFT software (Katoh et al., 2017). The resulting aligned sequences were entered into IQ-TREE version 1.6.7.1 (Nguyen et al., 2015) for construction of phylogenomic trees with maximum likelihood (ML) algorithms and 10,000 Ultrafast bootstrap approximation (Minh et al., 2013; Hoang et al., 2018). The best fit model for the ML tree was determined using the Bayesian information criterion by ModelFinder implemented in IQ-TREE (Kalyaanamoorthy et al., 2017). The resulting ML tree files were uploaded to iTOL (Letunic and Bork, 2019) for editing. Trees were presented as mid-point rooted trees.

## Results

### Assembly and Annotation of the *H. halys* Tissue Transcriptomes

Deep sequencing of the transcriptomes isolated from ASG, PSG, and gut of *H. halys* resulted in generation of 66.5 (PSG), and 81.7 (ASG) million single-end reads. The raw reads were trimmed and the resulting high-quality reads were used for assembly of transcripts. Transcripts from the ASG, PSG, and gut were assembled separately. The numbers of transcripts assembled (contigs) for each sample are shown in Table 1. The numbers of contigs encoding putative peptides of ≥100 aa were 22,185 (∼30% of total ASG contigs of >200 nt), 16,745 (42% of PSG contigs), and 20,240 (36% of gut-derived contigs). A summary of statistics for assembly of the transcriptomes is provided in Table 1.

**TABLE 1:** Summary of *H. halys* ASG, PSG, and gut transcriptome statistics.

**Tissue**	**ASG**	**PSG**	**Gut**
Total raw reads (million)	81.7	66.5	77.1
Reads after trimming (million)	72.3	61.5	71.7
Total no. of contigs (≥00 nt)	74,632	39,684	55,967
Total length (nt)	54,253,793	26,160,907	40,490,240
Mean length (nt)	654	659	723
N50	1,114	916	1106
No. annotated contigs	31,523	23,528	28,234
% annotated contigs	42	60	50
No. contigs encoding potential proteins (≥100 aa)	22,185	16,745	20,240
No of translated peptide sequences (≥100 aa)	28,724	20,466	25,226

**FIGURE 1:**
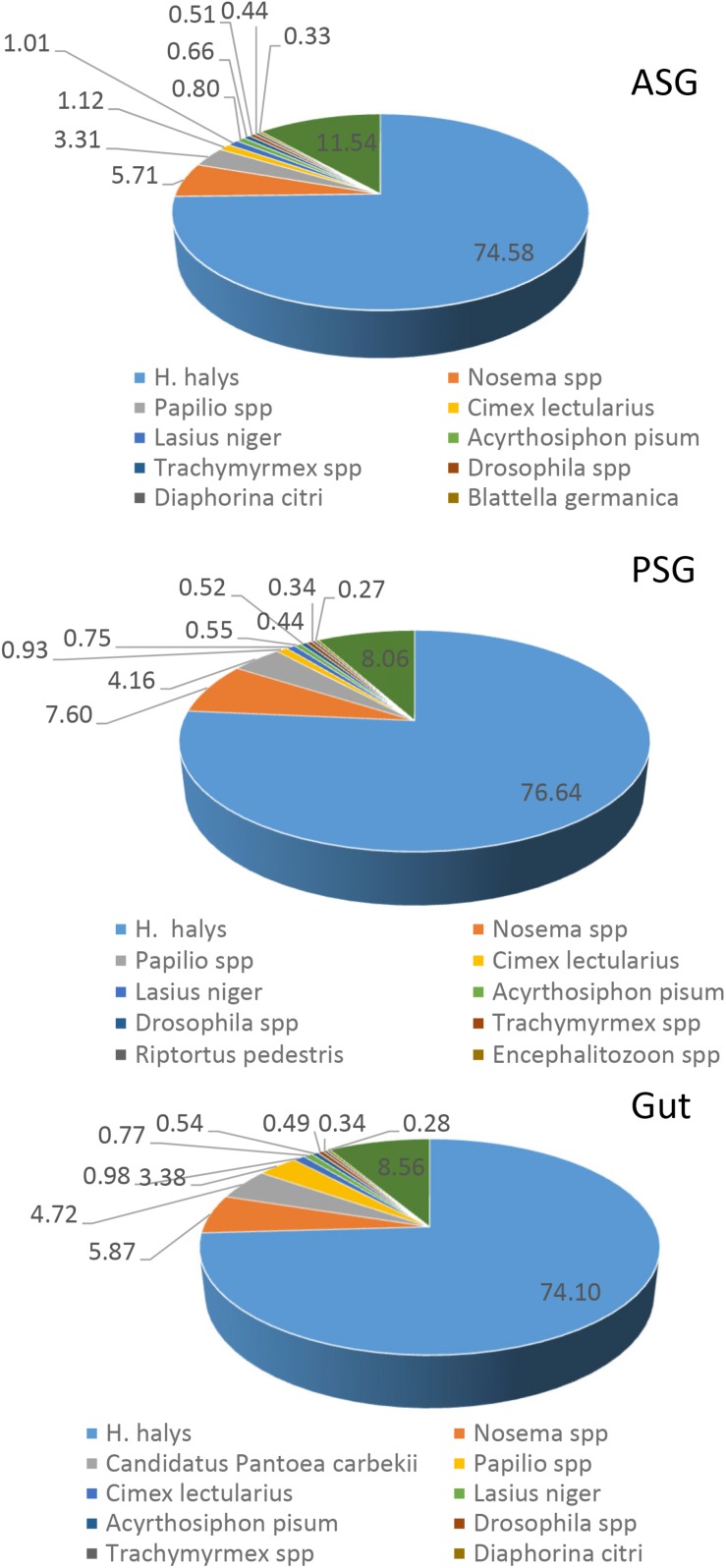
Species distribution of the best BLASTx hits in the nr database for ASG, PSG, and gut transcripts. The proportion (%) of sequences derived from specific species is shown for species with the highest number of hits. Some 75% of the top hit sequences were from predicted genes of *H. halys*. Transcripts derived from the symbiont *Nosema* were present in all three tissues, while transcripts of the gut-localized symbiont, *Candidatus Pantoea carbekii*, were present only in the gut.

**FIGURE 2:**
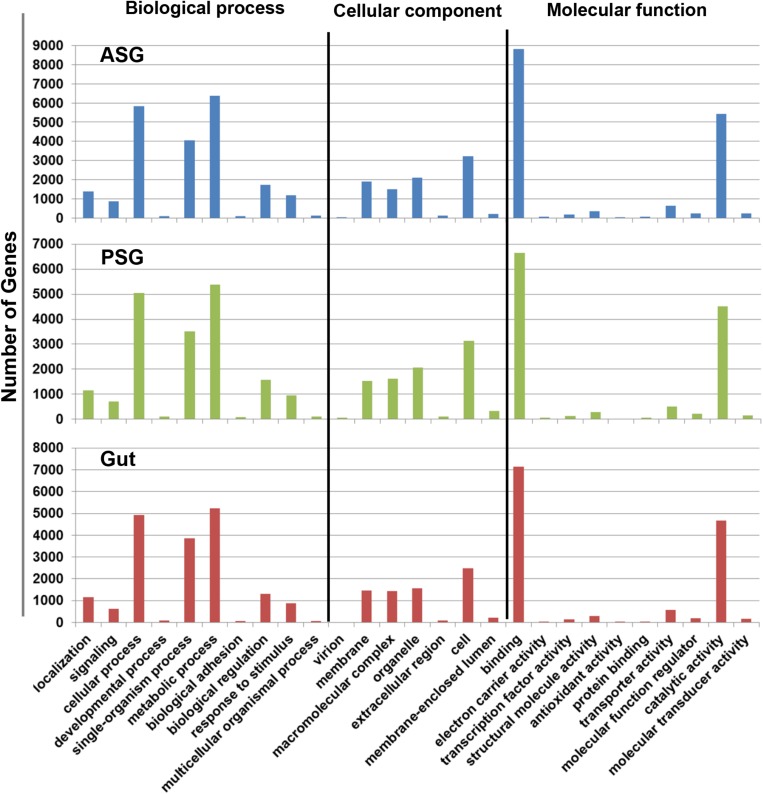
Gene ontology terms assigned to gene sequences involved in biological process, cellular component, and molecular function. Similar numbers of transcripts were assigned to each function for the ASG, PSG, and gut transcriptomes.

Initial annotation of the assembled transcripts was performed by BLASTx search against the NCBI nr database at an E-value of 1-e^–3^. The numbers of annotated contigs were 31,523 (42%) for ASG, 23,528 (59%) for PSG and 28,234 (50%) for the gut. The top hit sequences were derived from 746 species for ASG, 450 species for PSG, and 630 species for the gut transcriptome. As expected, the majority (>74%) of the transcripts hit predicted genes of *H. halys* (Figure 1). The E-values for *H. halys* hits were <1e^–20^ (data not shown). The proportion of transcripts for the top 10 species hit by *H. halys* transcripts are shown in Figure 1. For all three tissues, the organism with the second highest number of hits (5.7–7.6% of transcripts) was *Nosema*, a symbiont commonly associated with stink bugs (Sparks et al., 2014; Hajek et al., 2017). Transcripts that hit sequences of *Candidatus Pantoea carbekii*, a primary gut symbiont of *H. halys*, were only found in the gut transcriptome. Approximately 4% of the transcripts were derived from a *Candidatus* species (Figure 1). The Gene Ontology (GO) annotation, with transcripts grouped by functions of “Biological process,” “Cellular component,” and “Molecular function” is summarized in Figure 2. The annotated transcripts derived from the three tissues were comprised of similar numbers of GO terms in each functional category.

### Identification of *H. halys* Protease and Nuclease Transcripts

From the BLAST annotation results and sequence analysis we were able to identify unique transcripts of 234 putative proteases and 166 putative nucleases. The majority of the protease and nuclease transcripts identified were full- or near full-length. The proteases and nucleases identified are listed in Supplementary Tables S1, S2 respectively, along with relative levels of transcription (RPKM). A summary of the different categories of protease and nuclease transcripts identified in the *H. halys* tissues is presented in Table 2. Among the proteases, contigs of 44 aminopeptidases, 55 peptidases, 59 cathepsin-like/cysteine protease, and 48 trypsin-like/serine proteases were identified. In addition to the 211 protease sequences derived from *H. halys*, 23 proteases were apparently derived from symbionts of *Nosema* or *C. Pantoea* (including six genes that hit *Papilio xuths*) (Supplementary Table S1).

**TABLE 2:** Proteases and nucleases identified from *H. halys* transcriptomes.

**Enzyme**	**No. of top hits**			**Unique hits**	
	**Total**	***H. halys***	**Symbiont**	***H. halys***	**Symbiont**
**Protease**					
Aminopeptidase	44	31	13^∗^	28	10
Peptidase	55	50	5	46	3
Cathepsin-like/cysteine protease	59	59	0	54	0
Chymotrypsin	5	5	0	5	0
Trypsin-like/serine protease	48	48	0	45	0
Other putative protease	25	22	5	20	5
Total proteases	239	215	23	198	18
**Nuclease**					
Endonuclease	24	10	14	7	14
Exonuclease	20	11	9	8	9
Nucleotidase	20	18	1	10	1
Nuclease	21	16	5	8	5
Endoribonuclease	9	9	0	3	0
Exoribonuclease	19	11	8	8	8
Ribonuclease	53	38	13	25	13
Total nucleases	166	113	50	69	50

*^∗^Hits assigned to *Papilio xuthus* appear to be derived from *Nosema* spp.*

One hundred sixty-six putative nucleases were identified from the three transcriptomes (Supplementary Table S2). Of these, 113 of the contigs hit *H. halys* genes, 50 hit nucleases of symbionts, bacteria or microsporidia and three were from other insects. Fewer full-length sequences were acquired for putative nucleases from the transcriptomes, likely due to the lower levels of transcription relative to protease enzymes (Supplementary Table S2). Remarkably, 41% of the unique nuclease sequences appeared to be derived from symbionts, in contrast to 8.3% of the protease sequences that hit symbiont genes (Table 2).

### Mapping of *N. viridula* Proteomes to Predicted *H. halys* Protein Sequences

**FIGURE 3:**
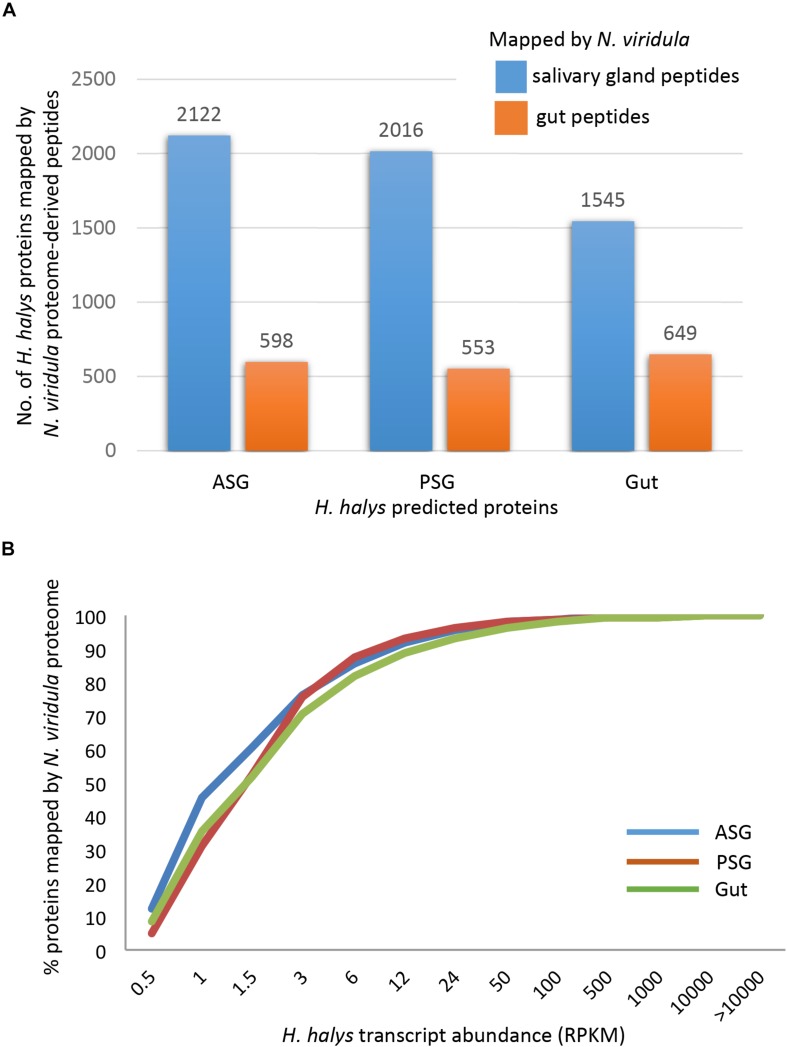
Mapping of peptides from the *N. viridula* gut and salivary gland proteomes to *H. halys* translated transcripts. *H. halys* transcripts were translated to protein sequences (≥100 aa). Translated protein sequences derived from the ASG, PSG, and gut were used as targets for mapping of peptides identified from *N. viridula* SG and gut (Lomate and Bonning, 2016). **(A)** Some 8–12% of the *H. halys* predicted protein sequences were mapped by peptides derived from the *N. viridula* salivary gland proteomes, while only 3% of the *H. halys* protein sequences were mapped by *N. viridula* gut-derived peptides. **(B)** Proteins translated from highly abundant *H. halys* transcripts, were more likely to be mapped by the *N. viridula* proteome. Translated protein sequences derived from the ASG, PSG, and gut were used as targets for mapping of peptides identified from *N. viridula* SG and gut (Lomate and Bonning, 2016).

The three sets of assembled *H. halys* tissue-derived transcripts were translated and the resulting protein sequences (≥100 aa) were used for mapping of proteome-derived peptide sequences. Proteomics libraries derived from the salivary gland (SG) and gut of *N. viridula* (Liu et al., 2018) were used for mapping. The proteomics profiles of *N. viridula* were useful for identification of *H. halys* proteins based on the high protein sequence identities between these two species. Peptide mapping results for the *N. viridula* proteomes are shown in Figure 3. From 8 to 12% of the *H. halys* predicted protein sequences were mapped by peptides derived from the *N. viridula* salivary gland (SG) proteome, while only 3% of the *H. halys* protein sequences were mapped by *N. viridula* gut proteins.

A total of 113 WS and 92 SS proteins mapped to the predicted protein sequences derived from the assembled *H. halys* transcripts, although some of the mapping results had low sequence coverage (Supplementary Tables S3, S4). Comparison of the proteins mapped by WS and SS peptides revealed that only 24 proteins were common to both WS and SS, with 89 and 68 proteins unique for WS and SS, respectively. The differences in the primary components of WS and SS likely reflect the respective biological functions of the WS and SS. The functions of 24 WS proteins and 22 SS proteins were unknown with either no hits or hits to uncharacterized *H. halys* proteins. The proteins common to the two salivary proteomes, many of which are involved in digestive processes, included amylases, carbonic anhydrases, chitinases, glycosidase, lectins, lipases, proteases, and nucleases. WS proteins included two proteins derived from *C. Pantoea*.

### Proteases and Nucleases Identified From *H. halys* Watery Saliva and Sheath Saliva Proteomes

Proteomics libraries derived WS and SS of *H. halys* (Peiffer and Felton, 2014) were next mapped to the *H. halys* predicted protein sequences. Putative proteases and nucleases identified from mapping of WS and SS peptides to predicted protein sequences derived from the assembled *H. halys* transcripts are listed in Table 3. In total, 22 proteases, one ribonuclease, and one potential nuclease were identified from the saliva of *H. halys*. Notably, no aminopeptidases were identified from either the WS or SS protein profiles. The proteases found in WS were peptidases (two carboxypeptidase B-like), cathepsin-like (two cathepsin L1-like), chymotrypsins (three) and trypsin-like serine proteases (14), while no chymotrypsin-like proteases were identified from SS. Only four (peptidase-5, trypsin-42, -45, -50) were found in both WS and SS, with 15 and three proteases being unique to WS and SS, respectively. Signal peptides were predicted for all of the proteases identified with complete N-terminal sequences, confirming secretion of these proteases from the salivary gland into saliva (Table 3). The RPKM values indicate that most of the enzyme transcripts with RPKM of >1000 were produced by the PSG, the exceptions being chathepsin-25 in the gut, and trypsin-48 in the ASG. Three proteases were transcribed at very high levels with RPKM > 10,000 with two (cathepsin-25 and trypsin-44) located in WS, and one (trypsin-45) in SS (Table 3). Surprisingly, only one nuclease (ribonuclease-31) and two uncharacterized nucleases (uncharacterized nuclease_f410 and uncharacterized nuclease_f435, which hit LOC106684787 LOC106684787/venom nuclease-like protein 1) (Supplementary Table S4), were identified from the WS of *H. halys* (Table 3).

**TABLE 3:** Protease and nuclease transcripts identified by mapping of *H. halys* watery saliva and sheath saliva proteomes to translated sequences.

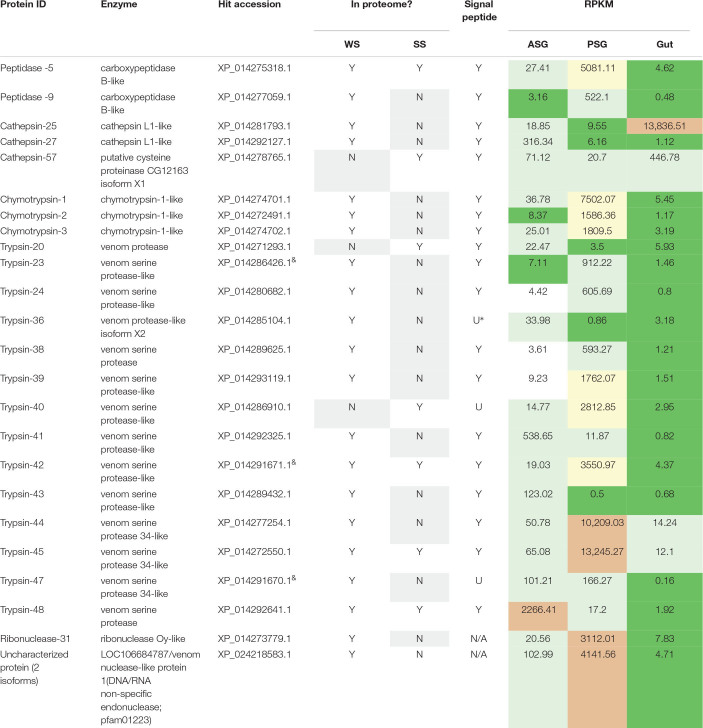

*The presence of each enzyme (protein) in watery saliva (WS) and sheath saliva (SS) is indicated, along with RPKM values shown in heat map format. RPKM values ≥10,000 are shown in orange; 1,000–9,999 in yellow; 10–999 in pale green; 0.1–9.9 in dark green. ^∗^Undetermined, missing N-terminal sequences. ^&^This record was removed from NCBI database as a result of standard genome annotation processing.*

### The Transcripts of Proteases and Nucleases Identified in the WS and SS Proteomes Were Highly Expressed

To determine relative transcript levels, the RPKM distributions of transcripts from each tissue (ASG, PSG, and gut) were determined. Similar RPKM distribution patterns were observed in the ASG, PSG, and gut transcriptomes: The RPKM values of ∼50% of the transcripts were less than 1.5, and ∼75% were less than 3, demonstrating that the majority of the transcripts were expressed at relatively low levels. In contrast, less than 2% of the transcripts had an RPKM of more than 100. Most of the proteases and nucleases had high RPKM in either PSG or ASG. The exceptions to this were cathepsin-25 and cathepsin-57, with high transcription levels in the gut (Table 3). Remarkably, the transcripts of 16 (67%) proteins had the highest RPKM in PSG. Seventy-five% of the identified putative enzymes had an RPKM of more than 500 in either PSG, ASG, or gut tissues. These results are consistent with our previous observation that proteins identified in the *N. viridula* tissue proteomes were derived from genes with high transcription levels (Liu et al., 2018).

### Comparison of Highly Transcribed Proteases and Nucleases in *N. viridula* and *H. halys*

*Halyomorpha halys* and *N. viridula* both belong to the family Pentatomidae and have highly homologous genes (Liu et al., 2018). To compare transcription of proteases and nucleases from these two species, we selected enzymes with the highest RPKM values of ≥100 for proteases and ≥20 for nucleases. In total 66 putative enzymes (54 proteases and 12 nucleases) were selected. The *N. viridula* counterparts of the selected *H. halys* proteins shared 60–97% sequence identities (Supplementary Table S5). The heat map of RPKM demonstrated that the vast majority of transcripts from the two stink bug species had similar transcription profiles (Supplementary Table S5). For example, peptidases, trypsins, and chymotrypsins were highly transcribed in the PSG, while cathepsins were primarily transcribed in the gut. Only a few chymotrypsins and trypsins showed moderate transcription levels in the gut for both *H. halys* and *N. viridula*. The only exception is *H. halys* cathepsin-53 which was highly expressed in ASG, but not in PSG or gut.

Nuclease transcription was lower overall than protease transcription levels, and nuclease transcription was generally higher in the ASG and gut tissues. Only ribonuclease-31 (ribonuclease Oy-like) was transcribed at a high level in the PSG and was detected in WS. Eight proteases and two nucleases identified in *H. halys* were not identified from *N. viridula* (Supplementary Table S5).

### Analysis of Proteases and Nucleases Identified From WS and SS

#### Peptidases

**FIGURE 4:**
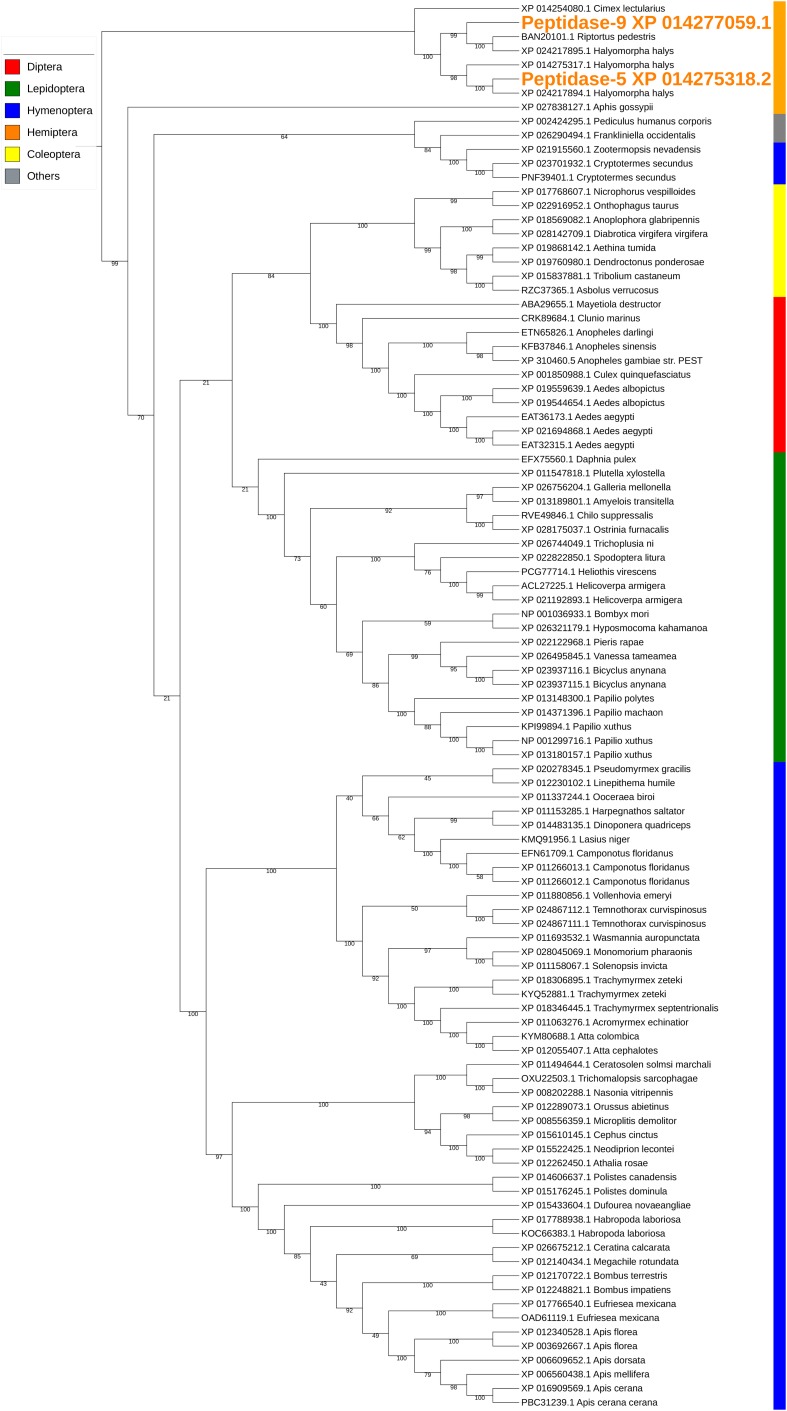
Phylogenetic analysis of amino acid sequences from peptidases found in both WS and SS. Peptidases-5 and -9 grouped with similar enzymes from other stink bugs and from bed bug. Insect orders are indicated by color as shown. Branch numbers are bootstrap values (%) of >40% (Bootstrap numbers of < 40% are not shown).

The two peptidases, peptidase-5/XP_014275318.2 and peptidase-9/XP_014277059.1, are both carboxypeptidase B-like with 44% sequence identity. Similar conserved domains, e.g., *propep_M14_superfamily* and *Peptidase_M14_like_superfamily* (domain accessions: *cd03860*, *smart00631*, *pfam00246*, *pfam002244*, and *COG2866*) were identified in these two peptidases. The transcription of peptidase-5 was nearly 10-fold higher than that of peptidase-9 in PSG (Table 3). Peptidase-5 was observed in both WS and SS, suggesting that this enzyme provides a primary function. Phylogenetically, the two peptidases group into the same clade, along with other peptidases from stink bugs and the bed bug (*Cimex lectularius*), and were distant from peptidases of other insects (Figure 4).

#### Cathepsins

**FIGURE 5:**
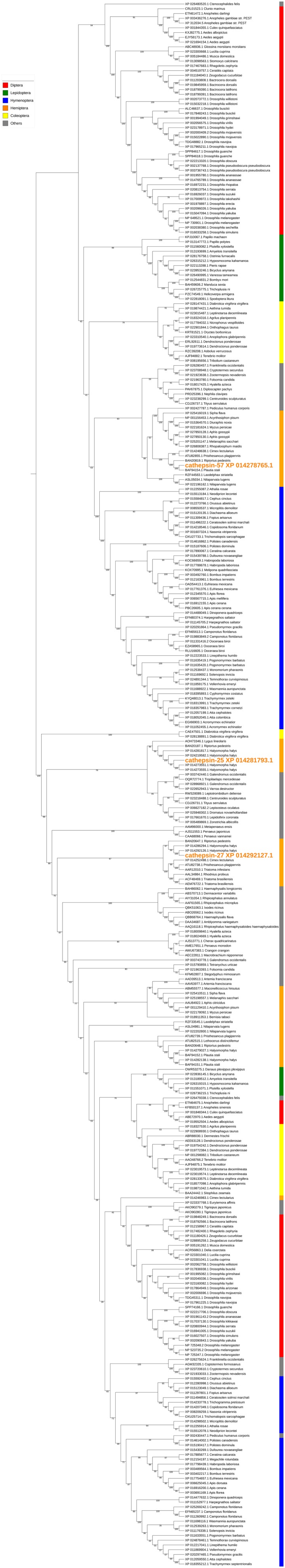
Phylogenetic analysis of amino acid sequences from cathepsins isolated from WS and SS. The positions of *H. halys* cathepsins−25, −27, and −57 in different clades are shown. Insect and “other” orders are indicated by color as shown. Branch numbers are bootstrap values (%) of >40%.

Two different types of cathepsin-like proteases were identified from the salivary proteomes. Cathepsin L1-like proteases (cathepsin-25/XP_014281793.1 and cathepsin29/XP_01492127.1) were identified from WS and cathepsin-57/XP_014278765.1, a putative cysteine proteinase CG12163-like was found in SS. Similar conserved domains (accessions: *pfam00112*, *cd02248*, *smart00645*, *PTZ00203*, *smart00848*, *pfam08246*, and *GOG4870*) were identified in both cathepsin-25 and cathepsin-27 proteins. In contrast to cathepsin L1-like cysteine proteases, cathepsin-57 contains multiple domains of the *CY superfamily* (accessions: *smart00043*, *cd00042*, and *pfam00031*) in addition to the domains found in cathepsin-25 and cathepsin-27. A phylogenetic tree based on selected sequences of arthropods showed two large clusters (cysteine protease CG12163-like and cathepsin -L1 like). In the cathepsin L1-like group, cathepsin-57, cathepsin-25, and cathepsin-27 were located on two separate branches (Figure 5). Cathepsin-25 was highly expressed in the gut, while cathepsin-27 was mainly expressed in ASG (Table 3). Differential expression of the two cathepsin L1-like proteases may reflect differences in function.

#### Chymotrypsins and Trypsins

**FIGURE 6:**
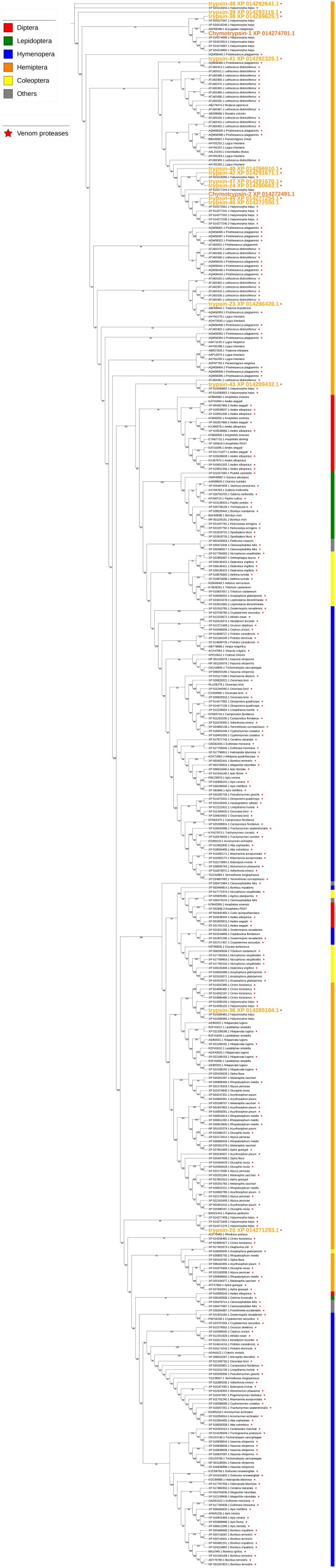
Phylogenetic analysis of amino acid sequences from trypsins and chymotrypsins isolated from WS and SS. Proteins assigned as “venom proteases” are indicated by ^∗^. Branch numbers are bootstrap values (%) of >40%.

Among the three chymotrypsins and 14 trypsins identified from WS and SS, chymotrypsin-1 and chymotrypsin-3 were highly homologous (sequence identity of 92%), and proteome peptide mapping did not distinguish between them (data not shown). Interestingly, three trypsins (trypsin-23, trypsin-42, and trypsin-47) were homologs of previously predicted trypsin genes of *H. halys* (XP_014286426.1, XP_014291671.1, and XP_014291670.1), but were later removed from the NCBI nr database. All identified chymotrypsins and trypsins contained common *Tryp_SPc superfamily* domains (accession: *cd00190*, *smart00020*, *pfam00089*, and *COG5640*). In addition, CLIP domain (*pfam1203* and *smart00680*), a regulatory domain in various trypsins, was present in two trypsins (trypsin-20/XP_014271293.1 and trypsin-36/XP_014285104.1), while CUB domain (*cd00041* and *smart00042*), an extracellular domain, was identified in trypsin-41/XP_014292325.1 and trypsin-43/XP_014289432.1. A phylogenetic tree based on selected trypsin sequences grouped the chymotrypsins and trypsins of WS and SS into two major clades of trypsin-like proteases from hemipteran insects. The chymotrypsins and a majority of the trypsins found in the saliva of *H. halys* were located in the same clade, with the salivary trypsins grouped into various sub-clades (Figure 6). In contrast, the two trypsins with CLIP domain motifs (trypsin-20 and -36) were located in a distant hemipteran clade.

#### Nucleases

**FIGURE 7:**
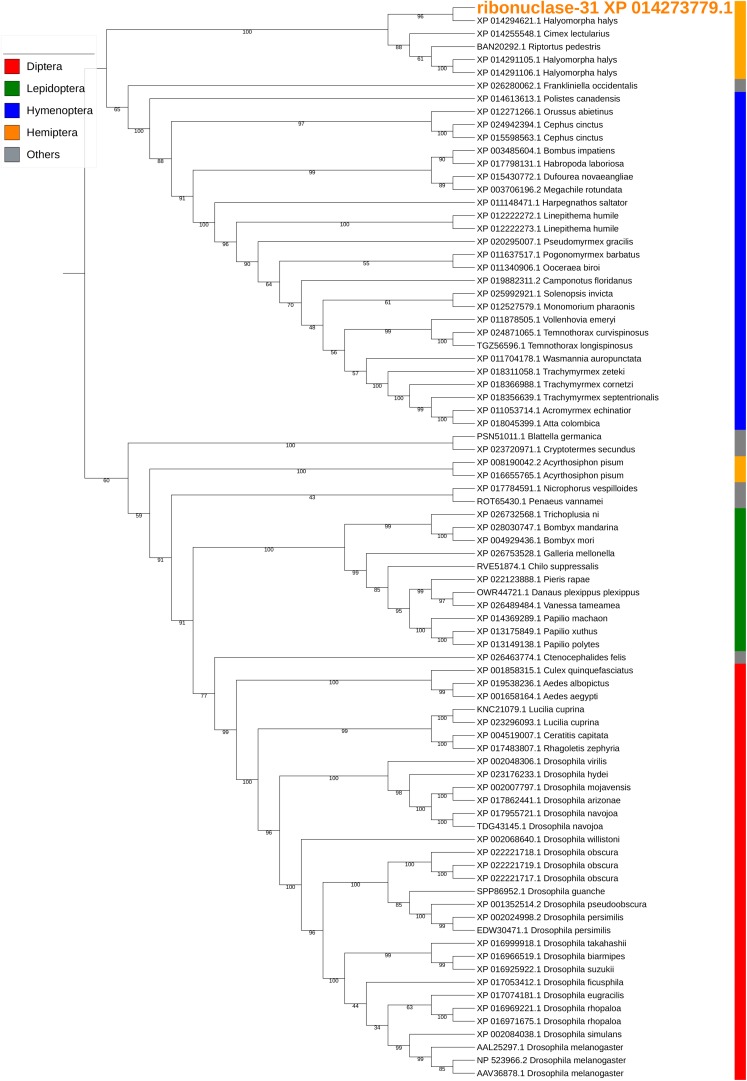
Phylogenetic analysis of amino acid sequences from ribonuclease-31. Ribonuclease-31 grouped with similar enzymes from other stink bugs and from bed bug. Insect orders are indicated by color as shown. Branch numbers are bootstrap values (%) of >40%.

Ribonuclease-31/XP_014273779.1 is a homolog of our previously reported SGSB_Ribonuclease-C20 (with 72% sequence identity) (Liu et al., 2018) and both genes were highly expressed in the PSG of *H. halys* and *N. viridula* (Supplementary Table S5). These ribonuclease Oy-like RNases contain *RNase_T2 superfamily* domains (*cd01961*, *pfam00445*, and *COG3719*), and may play an important role in host RNA degradation. The phylogenetic tree of ribonuclease-31 and homologous RNases of other insects showed that ribonuclease-31-like RNases group with similar RNases identified from stink bugs and the bed bug (Figure 7).

**FIGURE 8:**
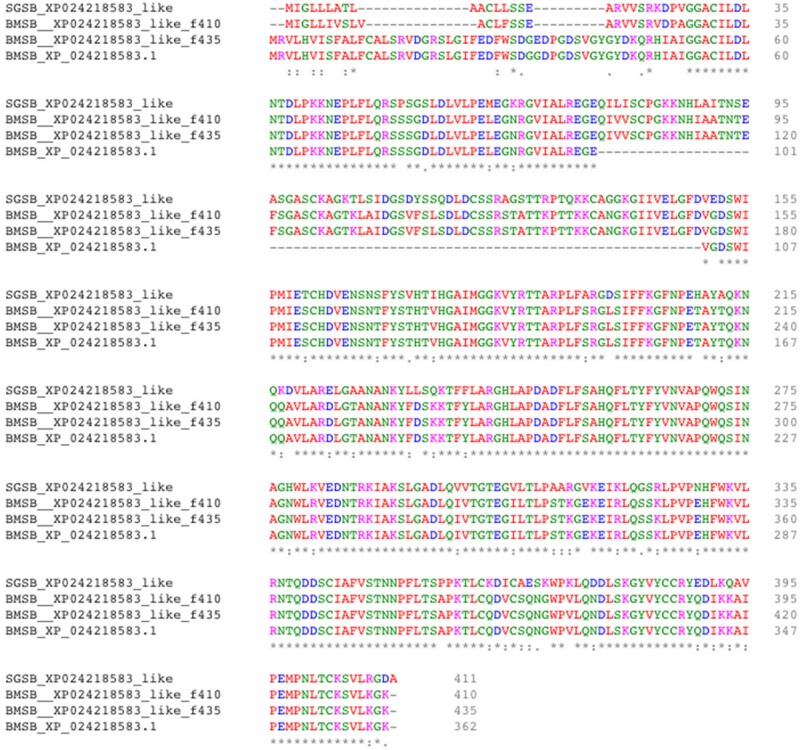
Alignment of XP_02421583.1 amino acids with homologous proteins from *H. halys* and *N. viridula.* Multiple pairwise sequence alignment was performed by use of the online Clustal Omega program (https://www.ebi.ac.uk/Tools/msa/clustalo/).

**FIGURE 9:**
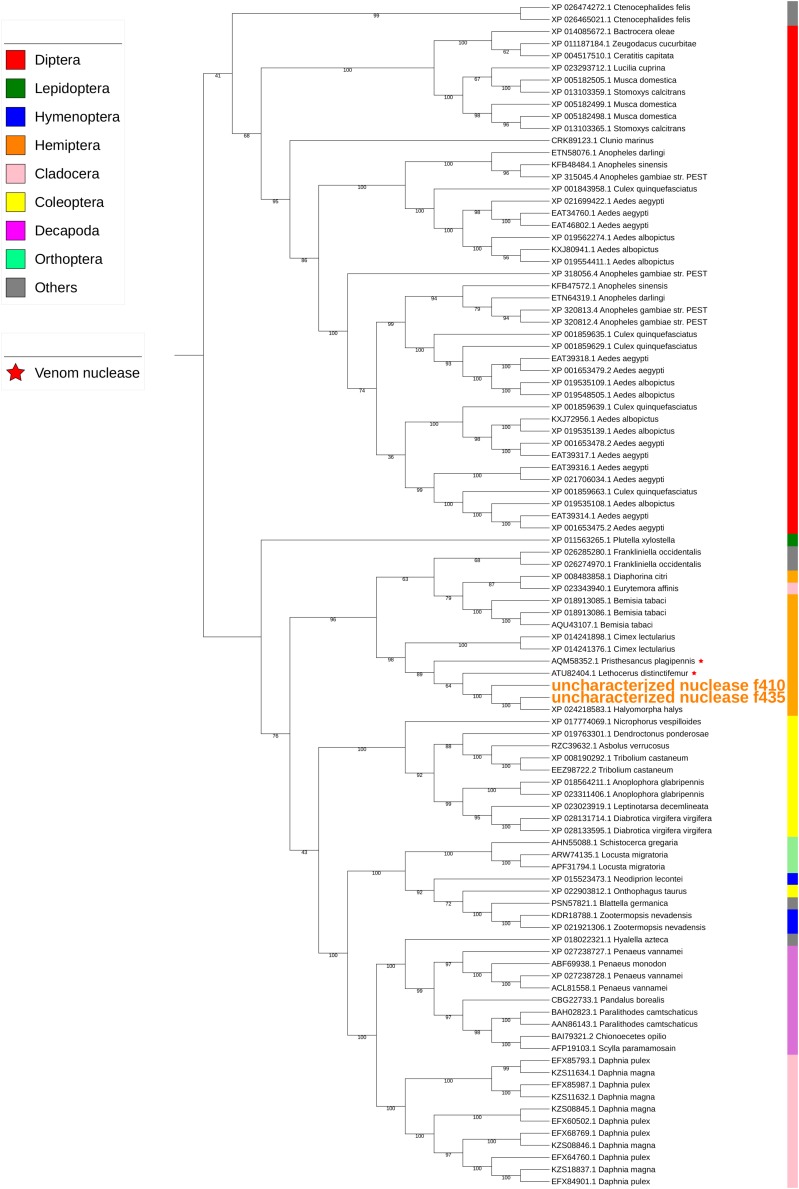
Phylogenetic analysis of amino acid sequences from uncharacterized XP_02421583.1-like nucleases. Uncharacterized nucleases f410 and f435 grouped with other hemipteran nucleases. Insect and crustacean orders are indicated by color as shown. Proteins assigned as “venom nucleases” are indicated by ^∗^. Branch numbers are bootstrap values (%) of >40%.

Two uncharacterized nucleases hit the LOC1064484787/XP_024218583.1 gene of *H. halys.* These two transcripts encoded 410 and 436 aa, longer than the predicted XP_024218583l.1 protein (362 aa). Proteins with homology to XP_024218583.1 have not previously been identified from *N. viridula* because the key word nuclease was missing from the BLAST annotation. On further analysis of *N. viridula* transcripts, we identified a homologous protein of XP_024218583.1 from the *N. viridula* transcriptomes of 411 aa, only one aa shorter than the uncharacterized nuclease_f410 and sharing 82% sequence identity. Protein sequence alignment of XP_024218583.1 with the three homologous protein sequences suggests that the predicted XP_024218583.1 was missing a 73 aa sequence (Figure 8). The sequence of the uncharacterized nuclease_f435 is identical to the predicted XP_024218583.1 sequence except for 73 aa missing from XP_024218583. The N-terminal sequence of the uncharacterized nuclease_f410 is similar to that of the XP_024218583-homologous protein of *N. viridula*, but different from the N-terminal sequences of the uncharacterized nuclease_f435 and XP_024218583 (Figure 8). These results suggest that two isoforms of XP_024218583 were transcribed in *H. halys*, and that the predicted XP_024218583.1 could be incorrect. Analysis of transcript abundance of the *N. viridula* version of uncharacterized nuclease_f410 (SGSB_XP_024218583_like) indicated that, similar to uncharacterized nuclease_f410, SGSB_XP024218583_like, it was highly expressed in PSG (RPKM: 3908 in PSG, 7.08 in ASG, and 2.2 in gut of *N. viridula*). The uncharacterized XP_024218583 like nucleases contain *NUC_superfamily* domains (*cd00091*, *pfam01223*, *smart00892*, *COG1864*, and *PTZ00259*), suggesting that they are DNA/RNA non-specific endonucleases that may function in digesting double- or single-stranded DNA and RNA. Similar to ribonuclease-31 (Figure 7), XP_024218583_like endonucleases of *H. halys* were closely related to those of other stink bugs, the bed bug and other hemipteran species (Figure 9).

## Discussion

The goals for this study were investigation of whether different phytophagous stink bugs employ common digestive enzymes, and to assess the relative roles of the ASG and PSG in production of salivary enzymes. From this work, we can draw the following conclusions: (1) *H. halys* produces at least 400 putative digestive enzymes (234 proteases, 166 nucleases) identified from the assembled sequences of the ASG, PSG, and gut transcriptomes. (2) More than 20 proteases and nucleases were identified from WS and SS and analysis of both proteomic and transcriptomic datasets indicated that the majority of proteases in WS were derived from the PSG. (3) The majority of the highly transcribed proteases and nucleases of *H. halys* were similar to those of *N. viridula* (Liu et al., 2018), indicating that phytophagous stink bugs employ a similar suite of proteases and nucleases for extra-oral and gut-based digestion.

Analysis of the ASG and PSG transcriptomes allowed for the identification of additional proteins present in the previously described *H. halys* WS and SS proteomes (Peiffer and Felton, 2014). The majority of digestive enzymes identified were present in the WS (19 proteases, 2 nucleases), with only 7 proteases found in SS. Of these 7 proteases, three were not identified in WS. Identification of these enzymes in the SS and WS proteomes implies functionality in extra-oral digestion. Although 44 putative aminopeptidases were identified from the ASG, PSG, and gut transcriptomes, none were found in the WS or SS proteomes. Among the two cathepsins (cathepsin-25 and -27) found in WS and cathepsin-57 detected in SS, cathepsin-25 and -57 were highly expressed in gut, suggesting that *H. halys* gut cathepsins can be delivered into saliva. There is a precedent for this suggestion: First instar *Tuberaphis styraci* aphid soldiers inject midgut-expressed cathepsin B-like proteases through their stylets into enemies, resulting in paralysis and death of the victims (Kutsukake et al., 2004). Similarly, the serine proteases detected in *H. halys* saliva were likely produced in the midgut and transferred to the saliva. The aminopeptidases of pentatomid stink bugs are highly expressed in the gut (Supplementary Table S1) (Liu et al., 2018), similar to other hemipterans (Cristofoletti et al., 2006). The majority of aminopeptidases are membrane-associated, which may explain why no aminopeptidases were found in WS and SS of *H. halys*.

Hemipteran SS was originally presumed to be produced by the PSG, while digestive enzymes were assumed to be produced by the ASG (Miles, 1972). If correct, the ASG would be the primary source of WS secretions. However, analysis of the *H. halys* WS and SS proteomes, ASG and PSG transcriptomes, and comparison with those of *N. viridula* (Liu et al., 2018) support a primary role for the PSG in production of enzymes (proteases and one nuclease) destined for the WS. Of the *H. halys* proteases and nucleases with RPKM of >1,000 (Table 3), eleven were produced in the PSG and one in the ASG. Of the 11 proteases highly transcribed in the PSG, all but one (trypsin-40) were found in WS and three were found in SS (trypsin-40, -42, -45). Trypsin-48, which was highly transcribed in the ASG, was detected in both WS and SS. These results indicate that enzymes produced by the PSG or ASG are not exclusively destined for the WS or SS, respectively. The different compositions of the SS and WS show that stink bugs are able to regulate the composition of their saliva.

Results from an ultrastructural analysis of the salivary glands of the Neotropical brown stink bug, *E. heros*, provide additional insight into the roles of the salivary gland tissues (Castellanos et al., 2017). Characteristics at the ultrastructural level suggest production of different compounds by the anterior and posterior glandular lobes of the PSG, muscle-mediated regulation of the mixing of these compounds, and control of the amount of saliva released from ASG and PSG at any given point during development (Castellanos et al., 2017). The ultrastructure of the ASG implicates this tissue in water transport and secretion but with limited storage capacity, implying that proteins synthesized are likely to be transported to the lumen of the PSG (Castellanos et al., 2017). The appearance of the PSG is typical of a tissue active in protein synthesis and secretion. The authors suggest that the anterior lobe of the PSG produces proteins for extra-oral digestion, while the posterior lobe produces other salivary components such as carbohydrates, lipids and other proteins. It follows that the ability of the stink bug to modify the composition of salivary components produced by the different tissues in the salivary gland facilitates the polyphagous habit of these insects. As the anterior and posterior lobes of the PSG were not separated prior to RNA extraction in the present study, we are unable to determine whether the digestive enzymes produced by PSG are primarily produced by the anterior lobe.

Interestingly, two proteins derived from the gut symbiont *C. Pantoea carbekii* were identified in the *H. halys* WS proteome. It is conceivable that these proteins were transported from the gut to the ASG, which appear to function in the transport of proteins from the hemolymph (Castellanos et al., 2017), and subsequently into the WS.

We previously observed that nuclease enzymes were abundant in *H. halys* saliva and salivary gland (Lomate and Bonning, 2018). In RNA-seq and proteomic analyses, a ribonuclease Oy-like RNase was highly expressed in the salivary glands of both *H. halys* and *N. viridula* (Liu et al., 2018), and was also identified in the WS proteome of *H. halys*. Ribonuclease-Oy-like RNase is a member of the RNase T2 family. T2 family RNases catalyze cleavage of single-stranded RNA, are found in a wide array of organisms (including protozoans, plants, bacteria, animals, and viruses) and have a broad range of functions (Luhtala and Parker, 2010). The other putative nuclease found from *H. halys* WS was an uncharacterized protein (LOC106684787; XP_024218583.1), which is an endonuclease_NS-like DNA/RNA non-specific endonuclease. A polyA binding protein (XP_013171827.1) was also detected in WS. As polyA binding protein is associated with mRNA turn-over (Mangus et al., 2003), this protein may also be involved in the degradation of host plant mRNA. It is hypothesized that nucleases secreted by stink bugs into the host plant function to degrade viral RNAs.

Many salivary proteases (trypsin-like) of *H. halys* hit proteases assigned as “venom proteases” by BLAST annotation. Phylogenetic analysis also indicated that salivary trypsins were related to “venom proteases” (Figure 6). Similarly, the uncharacterized nucleases-f410 and -f435 were closely related to two “venom nucleases” isolated from the assassin bug (a hemipteran predator), *Pisthesancus plagipennis*, and from the giant water bug or giant fishkiller, *Lethocerus distinctifemur* (Figure 9). Venoms from blood feeding insects and from insect predators, share features with the venoms of other organisms (Walker et al., 2016, 2017). While the composition of venom is complex, trypsin-like and chymotrypsin-like proteases are major venom components. Homologs of venom proteases are also found in plant-feeding hemipterans (Walker et al., 2016). It is unclear what role these “venom protease-like” trypsins and “venom nuclease-like” nucleases play following injection into plant hosts beyond potential functions in the degradation of host plant proteins and nucleotides.

## Conclusion

In conclusion, we have generated *H. halys* gut and salivary gland transcriptomes and identified the major proteases and nucleases produced by the ASG, PSG, and gut, along with those present in the WS and SS. The proteases and nucleases of *H. halys*, together with our previous characterization of proteases and nucleases from *N. viridula*, show that these phytophagous stink bugs encode and express similar suites of proteases and nucleases for extraoral digestion and gut-based digestion. Based on ultrastructural analysis, the differential mixing and release of salivary components from the ASG and PSG (anterior and posterior lobes) may mediate the ability of stink bugs to feed on multiple host plants (Castellanos et al., 2017). The comprehensive analysis of stink bug digestive enzymes presented here may provide leads for novel control strategies targeting digestive enzymes for management of multiple stink bug species, and highlight the common enzymatic challenges faced by bioactives in development for stink bug control.

## Data Availability Statement

The datasets generated for this study can be found in the NCBI Sequence Read Archive (SRA BioProject: PRJNA560285).

## Author Contributions

SL conducted the bioinformatics analyses. BB conceived the study and contributed to the design of the experiments. Both authors contributed to the writing and review of the manuscript.

## Conflict of Interest

The authors declare that the research was conducted in the absence of any commercial or financial relationships that could be construed as a potential conflict of interest.

## Footnotes

1 FASTQC https://www.bioinformatics.babraham.ac.uk/projects/fastqc/

2 FASTx-toolkit http://hannonlab.cshl.edu/fastx_toolkit/

3 BLAST2GO http://www.geneontology.org

4 Clustal Omega; http://www.ebi.ac.uk/Tools/msa/clustalo/

5 BioEdit https://softfamous.com/bioedit/

6 SignalIP 4.1 server http://www.cbs.dtu.dk/services/SignalP/

7 TransDecoder https://transdecoder.github.io

## Abbreviations

ASG: accessory salivary gland
BMSB: brown marmorated stink bug
PSG: principal salivary gland
SS: sheath saliva
WS: watery saliva.
